# P-1722. Establishment of the First Fungal Reference Laboratory in the UAE: Bridging Diagnostics, Resistance Surveillance, and Public Health Gaps

**DOI:** 10.1093/ofid/ofaf695.1893

**Published:** 2026-01-11

**Authors:** Fatima Al Dhaheri, Akela Ghazawi, Hari Vanam, Febin Anes, Fouzia Jabeen, Mushtaq Khan, Ahmed R Alsuwaidi, Jens Thomsen, Mohammad AlBataineh, Abiola Senok, Maya Habous, Anju Nabi, Hala Ismail, Carlo Kaabar, Connie Gibas, Stefan Weber, Nathan P Wiederhold

**Affiliations:** United Arab Emirates University, Abu Dhabi, Abu Dhabi, United Arab Emirates; United Arab Emirates University, Abu Dhabi, Abu Dhabi, United Arab Emirates; UAEU, Al Ain, Abu Dhabi, United Arab Emirates; UAEU, Al Ain, Abu Dhabi, United Arab Emirates; Purelab, Abu Dhabi, Abu Dhabi, United Arab Emirates; United Arab Emirates University, Abu Dhabi, Abu Dhabi, United Arab Emirates; United Arab Emirates University, Abu Dhabi, Abu Dhabi, United Arab Emirates; Medics Labor AG, Khalifa University (adjunct), Bern, Bern, Switzerland; Yarmouk University/George Washington (adjunct) University/Khalifa University (adjunct), Irbid, Irbid, Jordan; Mohammed Bin Rashid University of Medicine and Health Sciences, Dubai, Dubai, United Arab Emirates; Dubai Health, Dubai, Dubai, United Arab Emirates; Dubai Health, Dubai, Dubai, United Arab Emirates; Pure Lab, Sharjah, Sharjah, United Arab Emirates; Purelab, Abu Dhabi, Abu Dhabi, United Arab Emirates; UT Health San Antonio, San Antonio, Texas; Purelab, Abu Dhabi, Abu Dhabi, United Arab Emirates; University of Texas Health San Antonio, San Antonio, TX

## Abstract

**Background:**

The global rise in fungal infections and antifungal resistance highlights the urgent need for specialized diagnostic and surveillance capacity. Although fungal diseases are estimated to affect 2.5% of the UAE population, national laboratory infrastructure for fungal identification and resistance monitoring was previously absent. In 2024, the United Arab Emirates University (UAEU) established the country’s first fungal reference laboratory to address critical diagnostic gaps, support public health surveillance, and contribute to national AMR efforts.

**Methods:**

Between May 2024 and April 2025, a molecular mycology platform was launched, incorporating fungal culture, macroscopic/microscopic identification, internal transcribed spacer (ITS) and multilocus sequencing. A fungal biobank was created, linking isolates to clinical metadata. Transportation networks were developed across all Emirates for centralized processing. SOPs were adapted from international fungal centers. Core research areas included dermatophytosis, invasive molds, candidiasis, zoonotic fungi (One Health), and rare species discovery.

**Results:**

A total of 213 fungal isolates were collected; 130 (61%) were sequenced. Fungal identification turnaround time was reduced to under five days. Sequencing pipelines for molds such as *Aspergillus* and *Fusarium* were established. Dermatophyte surveillance showed 80% prevalence of terbinafine-resistant *Trichophyton indotineae* with Phe397Leu/Ala448Thr SQLE mutations. Pilot antifungal resistance surveillance and national stakeholder engagement were initiated to integrate fungal diagnostics into UAE AMR frameworks.

**Conclusion:**

The UAE’s first fungal reference lab has strengthened national diagnostic and resistance surveillance capacity. Future goals include scaling fungal biobanking, expanding susceptibility testing, and advancing One Health studies to position the UAE as a regional hub for fungal public health.

**Disclosures:**

Nathan P. Wiederhold, PharmD, Basilea: Grant/Research Support|bioMerieux: Grant/Research Support|Bruker: Grant/Research Support|F2G: Grant/Research Support|Mycovia: Grant/Research Support|Scynexis: Grant/Research Support|Sfunga: Grant/Research Support

